# Surgical approach in patients with T4 bladder cancer as primary treatment: Disaster or option with improved quality of life

**DOI:** 10.4103/0970-1591.38610

**Published:** 2008

**Authors:** Udo Nagele, Aristotelis G. Anastasiadis, Axel S. Merseburger, Jörg Hennenlotter, Markus Horstmann, Karl-Dietrich Sievert, Arnulf Stenzl, Markus A. Kuczyk

**Affiliations:** Department of Urology, Eberhard-Karls-University, Tübingen, Germany

**Keywords:** Bladder cancer, cystectomy, survival, treatment

## Abstract

**Objectives:**

Whereas local control is often insufficient in conservative management of T4 bladder cancer, neoadjuvant chemotherapy delays definite treatment, which could result in increased therapy-associated morbidity and mortality during the course of the disease. Primary cystectomy has been reported to be associated with a high complication rate and unsatisfactory clinical efficacy. Herein, we report postoperative outcome in 21 T4 bladder cancer patients subjected to primary cystectomy.

**Materials and Methods:**

Twenty-one patients underwent radical cystectomy for T4 (T4a/b: 14 and seven cases, respectively) bladder cancer. At the time of surgery, eight patients had regional lymph node metastases (N2: 6; N3: 2). The average age was 64 (52-77) years (≥70 years: n = 7). The postoperative follow-up was 13 (1-36) months for the whole group.

**Results:**

Mean duration of postoperative hospitalization was 19 (11-50) days. Whereas 10 patients received no intra - or postoperative blood transfusions, an average number of 3 (1-7) blood units were administered in the remaining cases. The mean postoperative hemoglobin value of patients not receiving any blood transfusions was 10 (8.5 - 11.4) g/dl. Major therapy-associated complications were paresthesia affecting the lower extremities (n = 3) as well as insignificant pulmonary embolism, enterocutaneous fistulation and acute renal failure in one patient, respectively. At the time of data evaluation, 11 patients were still alive after a follow-up of 20 (6-36) months. Four patients ≥70 years at the time of cystectomy were still alive 11, 11, 22 and 31 months following surgery, respectively.

**Conclusion:**

Primary cystectomy for T4 bladder cancer is a technically feasible approach that is associated with a tolerable therapy-related morbidity/mortality. Additionally, a satisfactory clinical outcome is observed even in a substantial number of elderly patients.

## INTRODUCTION

The gold standard for the treatment of muscle-invasive transitional cell carcinoma of the urinary bladder is radical cystectomy. However, the treatment of bladder cancer patients with locally advanced tumors classified as T4 disease remains a clinical challenge. Whereas the overall perioperative mortality rate irrespective of tumor stage has been reported to range between 2-5%[1] and to even increase with patients' age,[2] surgical treatment of advanced pelvic malignancies was documented to induce additional therapy-related mortality and morbidity.[3]

Therefore, different strategies have been developed to avoid surgery or to improve operability and/or postoperative clinical outcome of these patients. These approaches include both pure conservative multimodality strategies as well as neoadjuvant approaches in the form of chemotherapy alone or in combination with radiotherapy. However, in all these conservative therapeutic concepts of patients with bladder cancer extensively growing within the endopelvic region, a sufficient control of the primary tumor is rarely guaranteed. When salvage cystectomy becomes necessary due to severe complications, which result from an uncontrolled local situation, this procedure is likely to be associated with increased morbidity and significantly deteriorated clinical outcome. When the response to chemotherapy during a neoadjuvant concept is insufficient, further tumor growth can hamper the operability of the primary tumor. Additionally, a systemic chemotherapeutical treatment prior to surgery was reported to increase cystectomy-associated mortality.[1]

Considering the aforementioned remarks, we wanted to investigate the clinical outcome of 21 patients with T4 bladder cancer, who were subjected to primary radical cystectomy. One patient, who suffered a significant tumor progression and was included in a neoadjuvant treatment protocol, was excluded from the study. Clinical parameters such as the length of hospitalization, intraoperative transfusion rate, therapy-associated morbidity/mortality, the application of systemic chemotherapy prior to or following surgery as well as postoperative long-term survival were evaluated in all patients.

## MATERIALS AND METHODS

**Figures 1 and 2:**
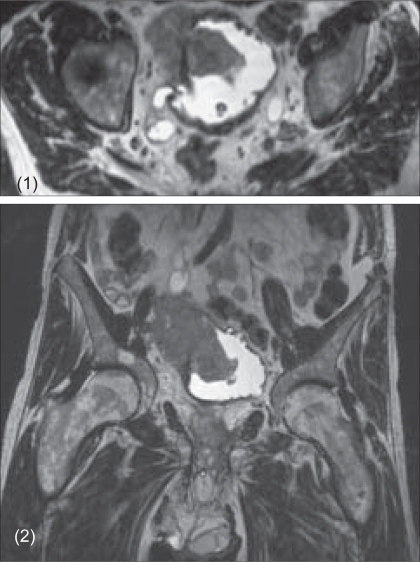
MRI of a pT4b TCC with consecutively dilated right ureter

Twenty-one patients underwent radical cystectomy for muscle-invasive, locally advanced bladder cancer between September 2002 and March 2005 in the Department of Urology, University of Tübingen, Germany. Detailed information regarding surgical treatment, transfusion rate as well as the peri- and postoperative morbidity and mortality were obtained from the patients' medical records. Perioperative mortality was defined as death within 30 days following surgery. The preoperative tumor staging included either CT or MRI scans [Figures 1-2] of the abdomen and chest as well as bone scan.

According to the histopathological evaluation of the cystectomy specimens, all tumors were classified as T4 disease (T4a/b: 14 and seven cases, respectively). While a solitary osseous lesion was observed in one case, no distant metastases became visible during preoperative tumor staging procedures in the remaining patients. At the time of surgical intervention, in eight patients lymph node disease (pN2: 6; pN3: 2) was either localized within the pelvis or above the aortic bifurcation. All but one patient (orthotopic ileal neobladder substitution) received an ileal conduit as urinary diversion. Though the average age of the patients was 64 (52-77) years, the age of seven (33%) patients was 70 years or older. The follow-up for the entire cohort of patients was 13 (1-36) months. One patient was subjected to surgery due to a tremendous tumor progression following administration of two cycles Cisplatin/Gemcitabine (Gemcitabine 1,000 mg/m^2^ on Days 1, 8 and 15; cisplatin 70 mg/m^2^ on Day 2) in a neoadjuvant setting. Four patients received four to six cycles of the identical chemotherapeutical regimen and three patients were subjected to radiation therapy postoperatively.

## RESULTS

At the time of data evaluation, 11 patients were still alive after a follow up-period of 20 (6-36) months (seven patients with pT4a [two of these patients received adjuvant chemotherapy] and four patients with pT4b tumors [one of these patients received radiotherapy]). Four patients ≥70 years at the time of surgery were alive after a follow-up period of 11, 11, 22 and 31 months, respectively. During follow-up, 10 patients died of progressive disease within a mean time interval of seven (2-19) months postoperatively; two patients had pT4aN0, two patients pT4bN0 and six patients with simultaneous lymph node metastases (four patients with pT4a, N2, G3, one patient with pT4a, N3, G3 and one patient with pT4b, N1, G2).

In one patient, a complete resection of the tumor specimen was not possible and in another three patients the histopathological evaluation of the cystectomy specimen revealed a positive margin resulting from extensive tumor growth on the level of the pelvic floor. Except one patient with an enterocutaneous fistula, who died of this complication, the remaining patients died of progressive disease during the follow-up period.

The mean duration of the postoperative hospitalization was 19 (11-50) days. Whereas 10 of the patients received no intra - or postoperative blood transfusions, an average number of 3 (1-7) blood units (1 unit: two patients; 2 units: four patients; 4 units: two patients; 5 units: one patient; 6 or 7 units: one patient, respectively) was administered in the remaining cases. The mean hemoglobin value of patients who did not receive intra - or perioperative blood transfusions was 10 (8.5-11.4) g/dl as detected immediately after surgery.

In addition to minor complications in the form of prolonged wound healing that were observed in four patients, other complications directly associated with surgery were mild paresthesia affecting the lower extremities (three patients) as well as insignificant pulmonary embolism and acute renal failure in one patient, respectively. In addition, one patient with an enterocutaneous fistula died of this complication three months after cystectomy.

## DISCUSSION

The clinical efficacy of a multimodality bladder-preserving strategy for the management of muscle-invasive bladder cancer has been repeatedly described to approximate that of radical cystectomy. Whereas this conservative approach might be a valid option for selected bladder cancer patients with tumors confined to the bladder wall,[4–7] it was described to induce severe local complications with a substantial negative impact on quality of life issues in non-selected groups of patients.[8–10] Lodde *et al.*, subjected 24 muscle- invasive bladder cancer patients who had refused or were not eligible for radical cystectomy to a multimodality bladder-preserving approach. With this therapy, all patients complained of urgency, frequency and severe nocturia. Whereas the mean readmission rate for the whole group was eight per patient, a salvage cystectomy became necessary in seven cases due to uncontrollable bleeding from the primary tumor. Additionally observed major complications were ileus, enterovesical fistulation and hydronephrosis from tumor-induced ureteral obstruction as well as symptoms occurring secondary to the development of distant metastatic spread. However, in the series by Lodde *et al.*, a transurethral resection (TUR) was combined with radiation in nine, chemotherapy in four and radiochemotherapy in only two cases, respectively.[10] A more favorable clinical outcome could have possibly been achieved following a combined modality approach that would have included TUR, radiation and chemotherapy. However, in contrast to this series, a complete TUR of the primary tumor is not possible in most patients presenting with extensive tumor growth beyond the bladder wall classified as T4 disease. A sufficient response to conservative treatment in the form of radio-chemotherapy can be hardly achieved in these cases.[11,12] Therefore, a clinical efficacy and complication rate similar to that reported by Lodde *et al.*, can be postulated for a conservative approach to bladder cancer revealing extensive endopelvic growth. Additional arguments that question the value of a conservative approach include increased mortality and morbidity following extensive chemotherapeutical pre-treatment when salvage cystectomy becomes necessary in case of severe local complications due to insufficient local tumor control.[13] In terms of the clinical prognosis, in one of the very rare series that compared conservative treatment of muscle-invasive bladder cancer with the clinical efficacy of radical cystectomy, salvage cystectomy was necessary in 25% of conservatively treated patients. Disease-free as well as long-term survival was significantly longer in the cystectomy group.[8] However, conservative management of T3/T4 bladder cancer has been reported to hardly exceed a median long-term survival of 27 months and an overall five-year survival rate of 19%.[5,14] With sufficient local tumor control achieved by cystectomy, 50% of patients included in the present series were still alive after a follow-up of 20 (6-36) months. A more extended follow-up period is necessary to compare the clinical prognosis of patients subjected either to surgical or conservative management of the disease. In contrast, the very limited clinical efficacy of salvage cystectomy following a conservative multimodality therapy has already been demonstrated.[11]

For neoadjuvant approaches in the form of radiochemotherapy or radiation alone, response rates range between 50 and 65%. A small survival benefit of about 5% has been described for bladder cancer patients subjected to neoadjuvant treatment within randomized clinical trials. However, in patients with gross endopelvic disease, it is questionable whether a similar long-term effect can be achieved. Although an improved operability as a result of a clinical downstaging has been described for the latter patients, the postoperative long-term survival, mainly of patients presenting with ≥T3 disease, remained unaffected in unselected cohorts.[15,16] Additionally, if the response to the neoadjuvant treatment is insufficient, the delay in cystectomy can cause uncontrolled tumor progress that prevents the achievement of a negative margin status by surgery. As a typical example, one of the patients included in our series presented with extensive local tumor growth and gross nodal disease (N3) following inclusion in a neoadjuvant treatment protocol. In the present study and in concordance with the literature,[17] the vast majority of patients died from progressive disease when a complete local resection was no longer possible.

In conclusion, the outcome of the present study demonstrates that primary cystectomy for the treatment of T4 bladder cancer is in general far away from a disaster, it is not only a technically feasible procedure, but also associated with a very tolerable therapy-related morbidity and mortality. Additionally, a satisfactory clinical outcome with prevention of local complications that significantly deteriorate quality of life issues can be achieved in a substantial number of even elderly patients subjected to a primary surgical treatment.
